# Conversational Flow Promotes Solidarity

**DOI:** 10.1371/journal.pone.0078363

**Published:** 2013-11-12

**Authors:** Namkje Koudenburg, Tom Postmes, Ernestine H. Gordijn

**Affiliations:** Department of Social Psychology, University of Groningen, Groningen, The Netherlands; University of Queensland, Australia

## Abstract

Social interaction is fundamental to the development of various aspects of “we-ness”. Previous research has focused on the role the content of interaction plays in establishing feelings of unity, belongingness and shared reality (a cluster of variables referred to as *solidarity* here). The present paper is less concerned with content, but focuses on the *form* of social interaction. We propose that the degree to which conversations flow smoothly or not is, of itself, a cue to solidarity. We test this hypothesis in samples of unacquainted and acquainted dyads who communicate via headsets. Conversational flow is disrupted by introducing a delay in the auditory feedback (vs. no delay). Results of three studies show that smoothly coordinated conversations (compared with disrupted conversations and a control condition) increase feelings of belonging and perceptions of group entitativity, independently of conversation content. These effects are driven by the subjective experience of conversational flow. Our data suggest that this process occurs largely beyond individuals' control. We conclude that the form of social interaction is a powerful cue for inferring group solidarity. Implications for the impact of modern communication technology on developing a shared social identity are discussed.

## Introduction

Audiovisual communication plays an increasingly important role in everyday human interaction. Notwithstanding the obvious efficiency advantages, modern technologies also make interactions susceptible to contextual disturbances, such as delays that subtly undermine the flow of the conversation. Because high-tech communication environments increasingly resemble face-to-face interactions, such delays may often go unnoticed by the speakers. Nevertheless, the present research suggests that even relatively small delays in mediated interaction can have quite powerful social effects: When conversational flow is disrupted by small delays, the development of solidarity is obstructed.

Research has shown that social interaction is a powerful social coagulant. Social interaction is fundamental to acts of social exchange and to the establishment of interdependence [1]–[3]. Moreover, social interaction is a forum for social comparison [4]–[5], and it is necessary for the emergence of socially shared realities, including the very notions of “I” and “we” [6]–[8]. Accordingly, social interaction also plays a role in the “bottom-up” induction of shared social identities: A feeling of we-ness in which a heightened sense of group entitativity, shared cognition and social identification are closely meshed [9]–[10]. In such interactive group settings, then, a sense of “we-ness” emerges that is characterized by feelings of unity, belongingness and shared reality which, although clearly distinct in some sense, are closely interrelated. For sake of convenience, we shall refer to these feelings as *solidarity* here. Explanations of the underpinnings of cooperation often treat the form of social interaction as subservient to the content. However, we propose that beyond these utilitarian and meaning-conveying functions, the shape of social interaction may also engender feelings of solidarity at a more basic, visceral level. This is because social interaction conveys a quality of coordination, which is in itself a key feature of solidarity.

Research on the form of social interaction has often focused on how interactions are smoothly and efficiently coordinated [11]–[13]. Research revealed that in interactions, people adjust linguistic, prosodic, and nonverbal features of their speech to match those of their partners and take into account the intentions and the (performed or prospective) actions of their partners in planning their own actions [13]–[14]. This interactive system allows people to exchange speech nearly continuously by taking turns, with minimal gaps in talk (0–500 ms) and minimal overlaps, creating a sense of conversational flow [15]–[17]. Having a conversation thus becomes comparable to other joint activities—such as dancing the tango, playing a duet, or shaking hands—in which coordination is the central, defining feature [11].

Research suggests that the source of conversants' ability to coordinate is their common ground: A set of knowledge, beliefs, and suppositions that speakers believe they share [11]–[12]. Such a framework of common understanding is often provided by groups to which people belong (e.g., [18]–[20]), suggesting that conversational flow is enhanced when speakers are members of the same group. But it is probable that this process is bidirectional [21]. Indeed, on the basis of our prior research [15], [22]–[23] we reason that the reverse process may be quite significant in social interaction. A conversation with good flow is a strong cue to the existence of common ground anchored in a sense of social unity and/or a positive relationship.

It follows that flow can be a precursor for the formation of a sense of solidarity or “we-ness,” between conversation partners. This process is central to the present research. Previous research has tended to focus on the content of interaction, or on the mere presence of interaction itself. For example, research on social identity formation suggests that the development of solidarity (in particular shared identity and shared cognition) is fueled by social interaction [10], focusing on the *content* of communication. In addition, interdependence research has shown that social interaction increases entitativity and cohesion [24], without clarifying whether content or form was responsible for this.

The present research, by contrast, isolates the influence of the *form* of communication on the development of solidarity from its content. We distinguished three aspects of solidarity that might be influenced by flow. Two of these are different sides to a sense of we-ness: “Cold” perceptions of group-level unity or “entitativity” [25] and “warm” feelings of group belonging. Entitativity is a gestalt psychological concept that refers to the degree to which an aggregate is perceived to be a unit. In the social identity tradition, feelings of entitativity can emerge because members of one group are contrasted to an outgroup [26]. In the interdependence literature, entitativity refers to the degree to which group members are interdependent and form a cohesive group [1], [27]. Belonging is a closely related concept (typically used in the literature on ostracism; e.g., [28]) that is associated with measures of social identification with the group (i.e., feelings of attachment to the group), but also includes items that consider the reverse direction of being accepted by the group. Thirdly, we examined experiences of *shared cognition*, a concept which is relevant to literatures on common ground, shared reality and social identity content [10], [29]–[30]. We expected that conversational flow signals the existence of common knowledge, values, and beliefs, which should be associated with feelings of social validation; or perceptions that one's beliefs are grounded, justified, and right [21], [31].

The second aim of this research was to explore whether the effects of conversational flow occur automatically—i.e., beyond individuals' control [32]. Interactions that are mediated by technology (e.g., video conferencing software, VOIP or even old-fashioned telephony) are prone to quite subtle disruptions in flow due to delays or technical deficiencies. We reasoned that making people aware of the source of such disruptions could provide them with the opportunity to consciously correct for any negative effects of disrupted flow, insofar as this might be possible. We therefore explored whether giving people the opportunity to attribute disruptions in conversational flow to factors beyond the communicators' control would reduce the influence of such disruptions on feelings of solidarity. To examine this, we conducted three studies in which participants had a conversation through auditory channels (Study 1–2) or audiovisual channels (Study 3, Fig. S1).

## Study 1

### Method

#### Ethics statement

The research was approved by the Ethical Committee Psychology of the University of Groningen. Informed consent was obtained in writing from all participants immediately before the research commenced.

#### Participants and procedure

Participants were 72 undergraduate students (57% female, 43% male; *M*age = 21.38 years, *SD* = 3.24), who participated in exchange for 5 euros. Participants were assigned to 36 dyads; the members of each dyad were unacquainted with each other. Participants occupied separate laboratory cubicles equipped with headsets. These headsets were connected to a computer on which the audio-recording program Record [33] was used for the interaction. Participants were instructed to have a 5-min conversation about holidays. To prepare for the conversation, participants were given a list of different holidays and asked to rate the extent to which they would like to go on each holiday (1* = not at all*, 7* = totally*).

To manipulate conversational flow, we randomly assigned dyads to either a flow or a disrupted-flow condition. In the flow condition, dyads had a 5-min conversation about holidays via headsets. The disrupted-flow condition was similar, except that the auditory feedback was delayed by 1 s throughout the second half of the conversation. Pilot research indicated that a 1 s delay was long enough to hamper the coordination of communicative behaviors and reduce the flow of the conversation without making participants consciously aware of the delay (cf., [34]).

#### Dependent measures

After the conversation, participants completed a questionnaire. Entitativity was measured with three items from the entitativity scale that we adjusted for dyads (α = .84; e.g. “I experience a sense of unity with the other participant” [9]). A three-item measure of feelings of belonging was derived from the Need Threat Scale (α = .81; e.g. “I had the feeling I belonged with the other participant” [28]). Additionally, a new scale of shared cognition was constructed based on prior scales for social validation [31] and shared cognition [21], and adjusted for use in dyads. This scale contained five items: “I had the feeling my partner and I were on the same wavelength”; “My partner and I understood each other”; “My partner and I agreed with each other”; “I had the feeling my opinions were validated”; and “I had the feeling my opinions were shared” (α = .92). Items for all measures were rated on scales from 1 (*strongly disagree*) to 9 (*strongly agree*). A manipulation check assessed the extent to which participants felt the conversation had flow (the Dutch word for flow is “*soepel*”, which conveyed the conversation proceeded smoothly and effortlessly).

### Results

The intraclass correlations for entitativity (.40), belonging (.59), and social cognition (.51) suggested that these scores were clustered within groups. To control for this nonindependence, we analyzed the data using multilevel modeling, with individuals (Level 1) nested in dyads (Level 2). Two outliers (standardized multilevel residual on one of the dependent variables >3) were removed.

We examined the effects of group-level flow on feelings of solidarity, which were measured at the individual level. The manipulation check indicated that participants in the flow condition experienced more conversational flow (*M* = 7.31, *SE* = .21) than did those in the disrupted-flow condition (*M* = 6.51, *SE* = .24), γ = 1.25, *SE* = .35, *t*(34) = 3.57, *p* = .001.

As predicted, participants in the flow condition felt more belonging, γ = .88, *SE* = .28, *t*(34) = 3.16, *p* = .004, *R* ^2^ = .42 and perceived their dyad to be more entitative, γ = .85, *SE* = .34, *t*(34) = 2.48, *p* = .02, *R* ^2^ = .31 than participants in the disrupted flow condition. No significant effect of flow on shared cognition was found, γ = .59, *SE* = .36, *t*(34) = 1.67, *p* = .10, *R* ^2^ = .16, although means were in the predicted direction (see Fig. S2). When including outliers in the analyses, similar results were obtained.

#### Mediation

We used the unconflated multilevel model approach [35]–[36] to examine whether the subjective experience of conversational flow was responsible for the effect on entitativity and belonging. A 2-1-1 multilevel mediation model was specified testing whether individual-level perceived flow mediated the effects of group-level manipulated flow on individual-level entitativity and belonging. The analyses revealed an indirect effect of flow via perceived flow on entitativity, γ = .62, 95% CI [0.15, 1.09], and on belonging, γ = .47, 95% CI [0.01, 0.92].

### Discussion

Study 1 showed that participants who had a conversation with flow experienced a higher sense of solidarity than did those who had a conversation in which flow was disrupted. In addition, the subjective experience of flow mediated the effects of manipulated flow on feelings of belonging and entitativity. However, results could reflect either the hypothesized elevation of we-ness in the flow condition or a decreased sense of we-ness in the disrupted-flow condition. We examined this possibility in Study 2 by modeling it after Study 1 but adding a control condition. Additionally, Study 2 explored whether individuals would be able to control for the effects of disrupted flow if they were made aware of the source of the disruption.

## Study 2

### Method

Participants were 130 undergraduate students (82% female, 18% male; *M*age = 19.86 years, *SD* = 2.24) who participated in exchange for partial course credits or 5 euros.

#### Procedure

First, unacquainted participants were assigned to dyads. As in Study 1, we assigned dyads to flow and disrupted-flow conditions, but we created two additional conditions. One was similar to the disrupted-flow condition, with one critical difference: Before starting the conversation, participants were informed that “the connection could be poor, due to which some glitches might occur.” This “cued” condition was intended to give participants an opportunity to attribute the flow disruption to technical deficiencies, and to examine whether this would reduce the effects of disrupted flow. The other new condition was a control condition in which participants were instructed to talk for 2 min about their holidays while the other member of their dyad listened but could not respond. After 2 min, these roles were switched. The control condition thus allowed dyads to exchange information similar to that exchanged by dyads in the other conditions, but in the absence of any actual conversation (or flow).

#### Dependent measures

We used the complete four-item entitativity scale (α = .91, [9]). Belonging was measured with four items derived from the Need Threat Scale (α = .86, [28]), excluding one item unsuitable for dyads (“I felt like an outsider during the conversation”). Shared cognition was measured as in Study 1 (α = .89).

### Results

Data were screened and analyzed as in Study 1. The intraclass correlations for entitativity (.10), belonging (.50), and social cognition (.30) indicated that multilevel analysis was required. Five dyads were excluded because they included a nonnative Dutch speaker, which could influence the flow of the discussion because of that member's difficulty with the Dutch language. Additionally, two outliers (standardized multilevel residual on one dependent variable >3) were excluded.

In order to systematically compare the four conditions, we used a Helmert contrast to compare each condition with all subsequent conditions. Thus, Ψ1 compared the control condition with the flow condition and both disrupted-flow conditions. Ψ2 compared the flow condition with both of the disrupted-flow conditions. Ψ3 compared the normal disrupted-flow condition with the cued disrupted-flow condition.

#### Manipulation check

The manipulation check confirmed that participants in the flow condition felt their conversations had more flow (*M* = 5.84, *SE* = .19) than did participants in the disrupted-flow condition (*M* = 5.27, *SE* = .14), Ψ2: γ = .53, *SE* = .20, *t*(56) = 2.69, *p* = .01. Additionally, participants in both the flow and the disrupted-flow conditions perceived the interaction to have more flow than did participants in the control condition, Ψ1: γ = .96, *SE* = .22, *t*(56) = 4.31, *p*<.001. The cue did not influence whether conversations were perceived as having flow, Ψ3: *t*<1, *ns*.

#### Dependent variables

As predicted, participants who had a conversation (the flow and disrupted-flow conditions) reported more belonging, γ = .68, *SE* = .17, *t*(56) = 3.98, *p* = .001, *R* ^2^ = .44; entitativity, γ = .63, *SE* = .19, *t*(56) = 3.38, *p* = .002, *R* ^2^ = .79; and shared cognition, γ = .80, *SE* = .17, *t*(56) = 4.86, *p*<.001, *R* ^2^ = .41 than those in the control condition did (Ψ1).

Moreover, as in Study 1, conversations with flow instigated more belonging, γ = .33, *SE* = .15, *t*(59) = 2.19, *p* = .03, *R* ^2^ = .21; and entitativity, γ = .37, *SE* = .17, *t*(56) = 2.22, *p* = .03, *R* ^2^ = .73 than conversations in which flow was disrupted (Ψ2). The data showed no significant effects of the flow manipulation on experienced shared cognition, γ = .21, *SE* = .15, *t*(56) = 1.41, *p* = .16, *R* ^2^ = .04, although effects were in the predicted direction (see Fig. S3).

No effect of the cue on any of the dependent variables was found (Ψ3: *t*s<.40, *ns*).

When including outliers and nonnative Dutch speakers in the analyses, the effects of Ψ2 on belonging and entitativity were smaller and achieved only marginal significance. The effects of Ψ1 did not change.

#### Mediation

As in Study 1 we examined whether the effect of flow (Ψ2) influenced the dependent variables via perceived flow. Ψ1 and Ψ3 were added as covariates. Results showed an indirect effect of flow via perceived flow on entitativity, γ = .14, 95% CI [0.002, 0.29], and on belonging, γ = .23, 95% CI [0.08, 0.39].

### Discussion

Replicating Study 1, Study 2 shows that belonging and entitativity are influenced by the delay manipulation. Mediational analysis shows that the manipulation decreases the subjective experience of flow, which leads to lower levels of belonging and entitativity. Moreover, having a conversation (as opposed to giving and hearing monologues) strongly predicts the emergence of a sense of solidarity, because it increases feelings of belonging and entitativity as well as socially shared cognition.

The data revealed no effect of providing participants with a cue to the source of the delay, on any of the dependent variables. Possibly, the cue was too subtle and did not increase participants' awareness of the delay in their connection. To reduce this potential ambiguity, we provided participants in Study 3 with feedback about their Internet connection during the conversation.

In addition, in Study 3 we sought to increase the generalizability of our findings from Studies 1 and 2 in three different ways. First, we conducted the study at a job fair to examine whether the findings from our studies conducted in a lab environment would replicate in a more naturalistic environment. Second, we had participants communicate via both auditory *and visual* channels, using computers. Finally, unlike the previous studies, we also included participants who were already acquainted with each other.

## Study 3

### Method

#### Participants and design

Participants were 134 individuals (60% female, 40% male; *M*age = 34.52 years, *SD* = 12.42, range = 17–61 years), who were recruited at a job fair to participate in a study about online interactions. Participants could participate either with an acquaintance (*n* = 78) or individually (*n* = 56). Those participating individually were assigned to a dyad. Dyads were allocated to one of four conditions that manipulated whether feedback was delayed throughout the second half of a 5-min conversation (disrupted flow vs. flow) and whether participants were provided with a cue about the connection (no cue vs. cue).

#### Procedure

Participants were informed that they would have a conversation with their acquaintance or another visitor of the job fair. If members of a dyad did not know each other, they were introduced briefly. Next, the two participants in each dyad were seated behind different tables with laptops, which were positioned so that direct visual or auditory contact was impossible. Here, participants filled out a questionnaire about holidays. Dyads were then instructed to have a 5-min conversation about holidays. Participants communicated via both visual and auditory channels using the laptops, which were connected by a network cable. A pretest in which we tested different durations of the delay in the video paradigm had indicated that 1.5 s was the most appropriate duration for reducing the subjective experience of flow. To make the delay as smooth as possible, without any visible glitches, we had it automatically set in after 2.5 min of conversation and slowly progress to a 1.5-s delay, which continued throughout.

To manipulate whether the delay could be attributed to a source other than the members of the dyads, we presented half of the participants with a cue about the Internet connection throughout the conversation. In the cued conditions, a bar was shown at the top of the screen displaying four little green squares accompanied by the text “CONNECTION IS GOOD”. In the cued disrupted-flow condition, at the moment the delay set in, these squares turned orange and the text “PROBLEMS WITH CONNECTION” was displayed. In the cued flow condition, the green squares and the text “CONNECTION IS GOOD” were displayed throughout the conversation. No information about the connection was given in the no-cue conditions.

#### Dependent variables

After the conversation, we had participants complete the same questionnaire used in Study 2 to assess their perceived flow (manipulation check), feelings of belonging, perceived entitativity, and shared cognition. In addition, participants rated their satisfaction with the experimental technology by indicating their agreement with seven statements, such as “I am satisfied with the quality of this program” using 7-point scales (1 = *completely disagree*, 7 = *completely agree*; α = .85). Finally, we asked participants whether they had known their interaction partner before the study (1 = *yes*, 2 = *no*).

### Results

Intraclass correlations for entitativity (.64), belonging (.42), and social cognition (.55) were high, indicating that multilevel analysis was required. Two dyads were excluded from the analyses because they included a nonnative Dutch speaker. Four outliers (multilevel standardized residuals >3 on one of the dependent variables) were removed from the analysis.

First, we examined whether the effects of conversational flow were replicated by regressing individual-level belonging, entitativity, and shared cognition onto group-level flow. Prior acquaintance of the members of dyads was added as a covariate in the analyses. Main effects showed that a priori acquaintance was related to higher levels of perceived flow (*M_acquaintance_* = 5.77, *SE* = .15, *M_stranger_* = 5.19, *SE* = .19), γ = .54, *SE* = .26, *t*(62) = 2.10, *p* = .039, *R* ^2^ = .13, satisfaction with technology (*M_acquaintance_* = 5.11, *SE* = .12, *M_stranger_* = 4.73, *SE* = .15), γ = .42, *SE* = .19, *t*(62) = 2.20, *p* = .032, *R* ^2^ = .11, entitativity (*M_acquaintance_* = 5.90, *SE* = .12, *M_stranger_* = 3.67, *SE* = .15), γ = 2.26, *SE* = .21, *t*(62) = 10.88, *p*<.001, *R* ^2^ = .80, belonging: (*M_acquaintance_* = 6.03, *SE* = .12, *M_stranger_* = 4.47, *SE* = .15), γ = 1.57, *SE* = .20, *t*(62) = 8.03, *p*<.001, *R* ^2^ = .84, and shared cognition: (*M_acquaintance_* = 6.02, *SE* = .09, *M_stranger_* = 4.89, *SE* = .11), γ = 1.17, *SE* = .16, *t*(62) = 7.35, *p*<.001, *R* ^2^ = .63. Testing the flow-by-acquaintance interaction revealed that the effect of the manipulation on perceived flow was larger among strangers than among acquaintances, γ = 1.22, *SE* = .50, *t*(61) = 2.46, *p* = .02. No other interaction effects were found, *t*s<1, *ns*. The effect sizes (*R* ^2^) that follow refer to the variance that is explained by the flow manipulation, as a percentage of the variance that remained after controlling for a priori acquaintance.

The manipulation check confirmed that participants in the flow condition perceived the conversation to have more flow (*M* = 5.89, *SE* = .18) than did participants in the disrupted-flow condition (*M* = 5.20, *SE* = .16), γ = .74, *SE* = .25, *t*(62) = 2.91, *p* = .005, *R* ^2^ = .32. Participants in the flow condition were also significantly more satisfied with the technology (*M* = 5.53, *SE* = .14) than were participants in the disrupted-flow condition (*M* = 4.37, *SE* = .13), γ = 1.15, *SE* = .18, *t*(62) = 6.23, *p*<.001, *R* ^2^ = .95. In addition, the findings from the previous studies were replicated: Flow increased perceived entitativity, γ = .46, *SE* = .20, *t*(62) = 2.25, *p* = .028, *R* ^2^ = .22, and belonging, γ = .39, *SE* = .19, *t*(62) = 2.02, *p* = .047, *R* ^2^ = .31, and marginally increased shared cognition, γ = .31, *SE* = .16, *t*(62) = 1.98, *p* = .052, *R* ^2^ = .12 (see Fig. S4A).

We next explored whether these effects were reduced when a cue was given as to the source of the flow disruption. To this end, the cue manipulation and the flow-by-cue interaction were added as predictors to the model. No extra variance was explained for entitativity, belonging, or satisfaction with the experimental technology, *R* ^2^<.03, and, moreover, neither the cue manipulation nor the flow-by-cue interaction significantly affected these variables (all *t*s<1.58, *ns*). For shared cognition, the cue manipulation and the flow-by-cue interaction explained extra variance, *R* ^2^ = .08. There was no evidence for a main effect of the cue factor (*t*<1, *ns*), but a marginally significant flow-by-cue interaction was found, γ = .29, *SE* = .16, *t*(62) = 1.85, *p* = .069 (see Fig. S4B). Further investigation of the interaction pattern suggested that among participants who were given no cue about their Internet connection, shared cognition was lower in the disrupted-flow condition than in the flow condition, γ = .90, *SE* = .44, *t*(62) = 2.07, *p* = .04. Among participants who were given a cue, shared cognition was not influenced by the flow disruption (*t*<1, *ns*). When including outliers and nonnative Dutch speakers in the analyses, similar results were obtained.

#### Mediation

The same analysis as used in Study 1 was performed, with level of prior acquaintance entered as a covariate. As in the previous studies, the analyses revealed an indirect effect of flow via perceived flow on entitativity, γ = .43, 95% CI [0.06, 0.80]), belonging, γ = .44, 95% CI [0.09, 0.79]), and shared cognition, γ = .36, 95% CI [0.07, 0.65].

Together, these results suggest that conversational flow predicts the emergence of a sense of solidarity. Additionally, providing participants with the opportunity to attribute the disruption of conversational flow to a deficient Internet connection did not reduce the effects of flow on feelings of belonging and entitativity. A marginal interaction effect suggested that the effects of flow on shared cognition were somewhat reduced as a result of introducing the cue.

## A Priori Consensus

The ratings of holidays that participants had made prior to the conversations in each of these studies provided an objective measure of a priori consensus within dyads. We examined whether the effects of conversational flow held when controlling for this baseline consensus. Results showed that in Studies 1 and 3, the effects of flow were not reduced when we controlled for a priori consensus. In Study 2, the effects were slightly reduced (See Additional Analysis S1 for a detailed description of these analyses).

## General Discussion

Three studies revealed that the subjective experience of conversational flow can lead to the emergence of a sense of we-ness. A similar pattern was found for the influence of perceived conversational flow on shared cognition, although the effects were clearly smaller. The effects of flow on entitativity, belongingness and shared cognition seem to occur independently of the content of the conversation. Moreover, these effects appear to be occurring automatically, in the sense that awareness that the disruption is occurring does not enable individuals to consciously compensate for the detrimental psychological effects of disrupted flow on feelings of solidarity.

These results highlight the importance of characteristics of conversation other than content for establishing solidarity. We believe that this is an important consideration in a world that is increasingly globalizing thanks to, for example, the spread of the Internet. Globalizing technologies not only facilitate communication but also introduce new forms of “high-bandwidth” social interaction (such as desktop video conferencing). As such new forms of social interaction become increasingly prominent means of conversation, they may ironically hamper the ability to establish particular kinds of social relations precisely because they do not allow people to realize the close coordination they expect a good conversation to have. Conversations can thus end up feeling “bad” for reasons that speakers do not understand. In such circumstances, technology may subtly undermine the emergence of solidarity.

Among others, this research has practical relevance for the design of communication technology. In the literature on technology-mediated audio and video interaction, there is a pervasive belief that face-to-face interaction is superior for many different purposes (e.g., [37]) and accordingly technology design tends to assume that it would be important for mediated communication to mimic “real” face-to-face interaction as much as possible [38]. The present research points to a specific social-psychological process that may explain one reason why such mediated communications that are “almost real” may nevertheless feel different and sometimes perform less well than expected. Whereas good conversational flow through instant interaction gives communicators the ability to form strong social bonds “inductively” (see also [39]), even very short delays can disrupt this process (especially in novel relationships) and thereby undo some of the supposed benefits of instant interaction. This suggests that some of the supposed negative social consequences of mediated communication may not be due to the limited bandwidth of technology *per se*, but rather to the suboptimal transmission of signals due to delays on the “line.” It follows that in future research on the effects of mediated communication in comparison with face-to-face interaction, it is essential for researchers to ensure that delays cannot be a confounding factor that may offer an alternative explanation for the results.

A broader theoretical implication of this research is that communication's social effects may stem from the act and art of conversation per se: The micro-level situation and dynamic are key factors that contribute to the emergence of higher order (macro-level) social processes and structures (see also [40]–[41]). Accordingly, the findings are relevant to the question of how “healthy” social relationships can be maintained across a wide variety of face-to-face settings: We believe that it would be important to pay close attention to the form of interaction in settings as varied as close relationships, work settings, education, and clinical settings. The present research adds the insight that the smooth taking of turns is an important aspect of the art of conversation, which may have significant consequences.

The idea that communication is a vehicle for social exchange is ancient in science and popular culture: In the biblical story of the Tower of Babel, God ends a state of solidarity among people by introducing multiple languages: “And from thence did the Lord scatter them abroad upon the face of all the earth” (Genesis 11∶9, King James Version). Our research suggests that although such social disintegration can result from the drastic step of creating multiple languages, it can also be achieved by more subtle and less discernible means. If one wanted to go to less trouble in undermining the world's unity, one could start with a dodgy internet connection obstructing conversational flow.

## Supporting Information

Figure S1 **Experimental setup.** Communication occurred via auditory channels (Studies 1 & 2) or audiovisual channels (Study 3).
(TIF)

Figure S2 **Mean levels of entitativity, belonging and shared cognition per condition of flow in** **Study 1** **.** Error bars represent standard errors.
(TIF)

Figure S3 **Mean levels of entitativity, belonging and shared cognition per condition in** **Study 2** **.** Error bars represent standard errors.
(TIF)

Figure S4 **Estimated marginal means for entitativity and belonging per condition of flow in** **Study 3** **.** Means are corrected for prior acquaintance. Error bars represent standard errors. A. Main effects of flow on entitativity and belonging. B. Cue-by-flow interaction on shared cognition.
(TIF)

Additional Analysis S1 **The role of a priori consensus.**
(DOCX)
