# Supplementary figures and images for: Conversational Flow Promotes Solidarity
Source: PLoS One. 2013 Nov 12;8(11):e78363. doi: 10.1371/journal.pone.0078363 (PMC3827030; doi:10.1371/journal.pone.0078363)

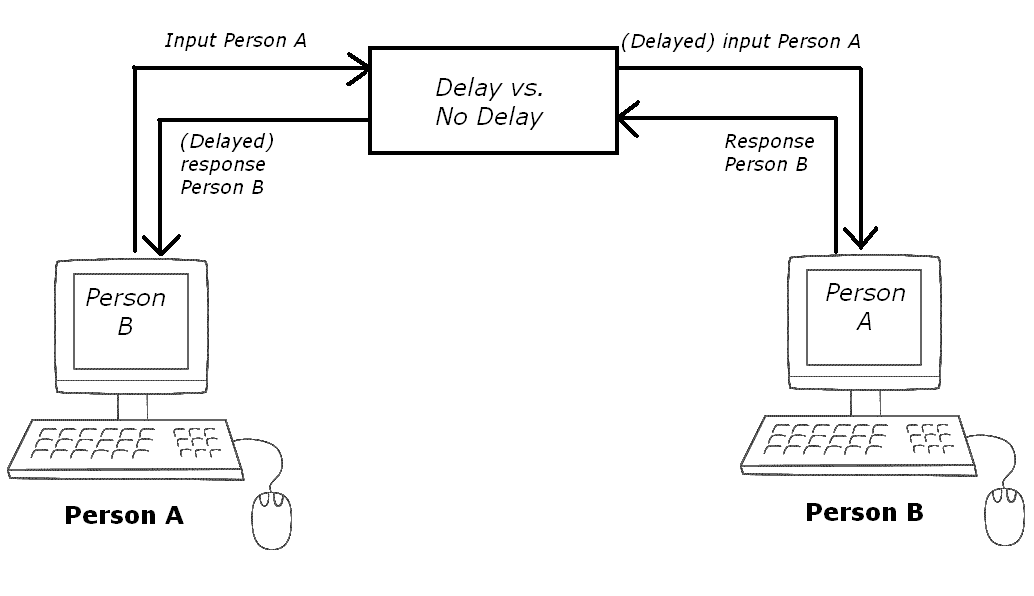

Supplement: Figure S1 — Experimental setup. Communication occurred via auditory channels (Studies 1 & 2) or audiovisual channels (Study 3). (TIF) [file pone.0078363.s001.tif]

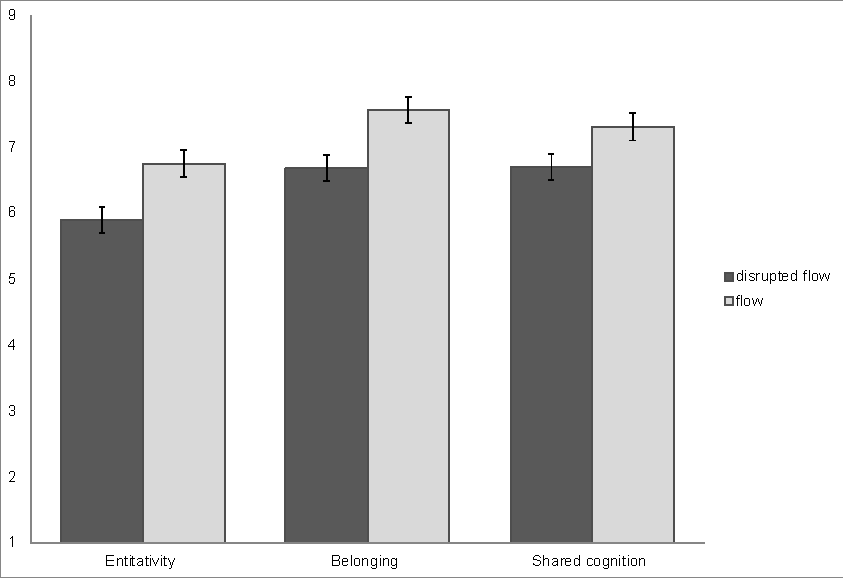

Supplement: Figure S2 — Mean levels of entitativity, belonging and shared cognition per condition of flow in Study 1 . Error bars represent standard errors. (TIF) [file pone.0078363.s002.tif]

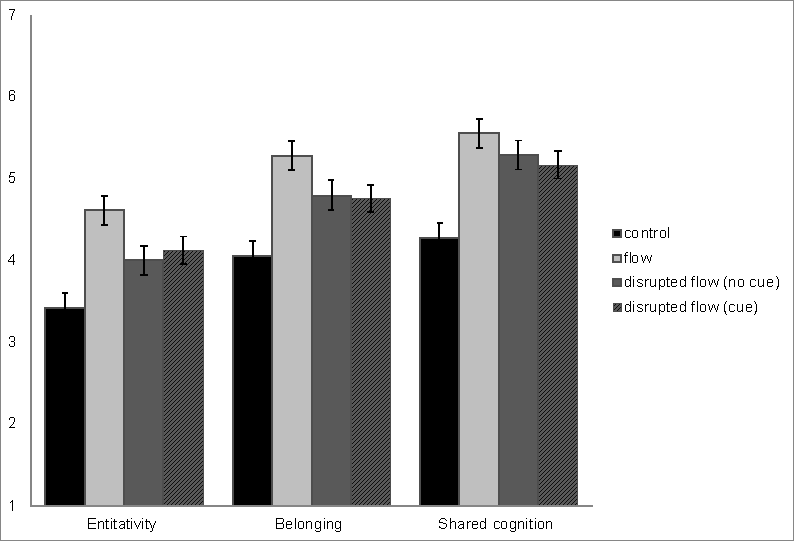

Supplement: Figure S3 — Mean levels of entitativity, belonging and shared cognition per condition in Study 2 . Error bars represent standard errors. (TIF) [file pone.0078363.s003.tif]

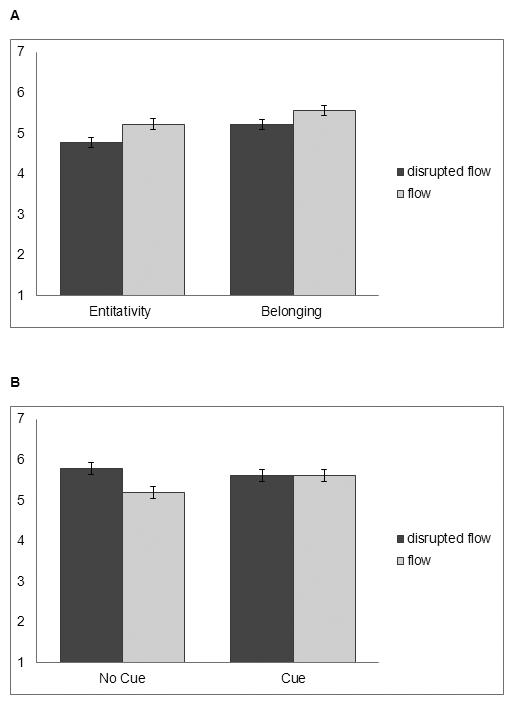

Supplement: Figure S4 — Estimated marginal means for entitativity and belonging per condition of flow in Study 3 . Means are corrected for prior acquaintance. Error bars represent standard errors. A. Main effects of flow on entitativity and belonging. B. Cue-by-flow interaction on shared cognition. (TIF) [file pone.0078363.s004.tif]
