# Supplementary material for: Conversational Flow Promotes Solidarity
Source: PLoS One. 2013 Nov 12;8(11):e78363. doi: 10.1371/journal.pone.0078363 (PMC3827030; doi:10.1371/journal.pone.0078363)
Supplement: Additional Analysis S1 — The role of a priori consensus. (DOCX) [file pone.0078363.s005.docx]

Supplementary Materials

**Additional Analyses: The Role of A Priori Consensus**

By using participants’ ratings of different holiday types and destinations, it was possible for us to calculate levels of consensus between members of each dyad prior to their conversation. In each study, before starting the conversation, participants in each dyad were given a list of eight types of holidays and seven holiday destinations and were asked to indicate the extent to which they would like to go on each type of holiday and to each destination, using 7-point scales (1 = *not at all*, 7 = *totally*). The holiday types were a “sun, sea, and beach holiday,” a “party holiday,” a “winter sports holiday,” a “city trip,” a “backpacking holiday,” an “excursion,” a “camping holiday,” and a “cruise holiday.” The different destinations were Australia, southern Europe, Scandinavia, Latin America, North America, Asia, and Africa. The levels of prior consensus within the dyad were assessed by averaging within-dyad differences in scores across all holiday types and destinations (averages standardized and multiplied by –1).

**Study 1.** To determine whether the effect of disrupted conversational flow on feelings of solidarity would hold when taking into account dyadic partners’ consensus prior to the conversation, we added the consensus scores to the model as a predictor. Using hierarchical linear modeling, the effects of the group-level flow (flow vs. disrupted flow) and a priori consensus were modeled to predict feelings of solidarity on the individual level. The data showed no influence of a priori consensus on feelings of shared cognition, γ = 0.04, *SE* = 0.19, *t*(33) = 0.20, *ns*, entitativity, γ = 0.05, *SE* = 0.03, *t*(33) = 1.59, *p =* .12, or belonging, γ = 0.03, *SE* = 0.14, *t*(33) = 0.19, *ns*. However, flow still positively predicted entitativity, γ = 0.86, *SE* = 0.36, *t*(33) = 2.43, *p =* .02, and belonging, γ = 0.87, *SE* = 0.29, *t*(33) = 3.00, *p =* .006. No significant effect of flow on shared cognition was found, γ = 0.61, *SE* = 0.37, *t*(33) = 1.65, *p =* .11, although means were in the predicted direction. These results reveal that conversational flow more strongly predicted the emergence of feelings of solidarity than did prior levels of consensus between members of dyads.

**Study 2.** Five participants did not fill out the questionnaire about holidays, so the level of consensus between them and their dyadic partners could not be estimated. Results showed that when controlling for consensus between interaction partners, having a conversation (as opposed to the control condition, ψ1) still strongly influenced participants’ feelings of belonging, γ = 0.66, *SE* = 0.20, *t*(47) = 3.33, *p =* .002, entitativity, γ = 0.65, *SE* = 0.22, *t*(47) = 3.01, *p =* .005, and shared cognition, γ = 0.80, *SE* = 0.19, *t*(47) = 4.22, *p <* .001.

However, flow (as opposed to disrupted flow, ψ2) now marginally predicted belonging, γ = 0.31, *SE* = 0.17, *t*(47) = 1.78, *p =* .08, and no longer affected entitativity*,* γ = 0.23, *SE* = 0.19, *t*(47) = 1.21, *p =* .23, or shared cognition (*t* < 1, *ns*).

A priori consensus influenced feelings of shared cognition, γ = 0.08, *SE* = 0.03, *t*(47) = -2.02, *p =* .05, but had no effect on feelings of entitativity, γ = –0.05, *SE* = 0.03, *t*(47) = –1.59, *p =* .12, or belonging, γ = –0.04, *SE* = 0.03, *t*(47) = –1.48, *p =* .14.

**Study 3.** One participant did not complete the questionnaire about holidays, so consensus could not be estimated for that participant’s dyad. When controlling for a priori consensus, the effects of flow on entitativity, γ = 0.42, *SE* = 0.20, *t*(60) = 2.07, *p =* .04, belonging, γ = 0.39, *SE* = 0.18, *t*(60) = 2.15, *p =* .03, and shared cognition, γ = 0.31, *SE* = 0.15, *t*(60) = 2.10, *p =* .04, remained significant. There was no evidence for an effect of prior consensus on any of these variables (all γs < 0.08, *t*s < 1.02, *ns*).

In addition, we found no main effects of the cue manipulation on any of the variables (all *t*s < 1.37, *ns*), whereas the effect of the flow-by-cue interaction on shared cognition remained marginally significant, γ = 0.54, *SE* = 0.30, *t*(60) = 1.76, *p =* .08. No other interaction effects were found (all *t*‘s < 1.13, *ns*).

Together, these results reveal that the effects of conversational flow on feelings of belonging and entitativity occur largely independently of the content of the conversation. Only in Study 2, taking into account a priori consensus between members of dyads led to a reduction in the effects of flow. However, in Study 1 and 3, the effects of flow on feelings of we-ness remained after taking into account a priori consensus.
